# Anatomical Rooms

**Published:** 1875-10

**Authors:** 


ANATOMICAL ROOMS—Plan for the Construction, Ventilation and Hygenic Management. By H. Lenox Hodge, M.D., Dem· Anat. University of Pennsylvania. Pamphlet; pp. 10.

